# Supplementary material for: The validity and reliability of motion analysis sensor system for wheelchair users (MASSWU)
Source: PLoS One. 2025 Oct 24;20(10):e0333391. doi: 10.1371/journal.pone.0333391 (PMC12551845; doi:10.1371/journal.pone.0333391)
Supplement: S1 File — (DOCX) [file pone.0333391.s001.docx]

**MASSWU Codes**

**// Main.cpp**

#include "main.h"

#include "setup.h"

#include "sensor.h"

#include "comm.h"

// Function to Setup The Sensor

void setup(void) {

// Initialize Sensor

setup_init();

// Create Queue

queue = xQueueCreate(10, sizeof(uint16_t));

// Create Task

xTaskCreate(sensor_task, "SENSOR", 2048, NULL, 2, NULL);

xTaskCreate(comm_task, "COMM", 2048, NULL, 1, NULL);

}

// Function to Loop The Sensor

void loop(void) {

vTaskDelay(1000);

}

**// Setup.cpp**

#include "main.h"

#include "setup.h"

BLEDfu bledfu; // OTA DFU service

BLEDis bledis; // device information

BLEUart bleuart; // uart over ble

BLEBas blebas; // battery

LSM6DS3 imu( I2C_MODE, 0x6A );

bool dataflag = false;

// Function to Initialize BLE

void setup_init(void){

Serial.begin(115200);

Bluefruit.autoConnLed(true);

Bluefruit.configPrphBandwidth(BANDWIDTH_MAX);

Bluefruit.begin();

Bluefruit.setTxPower(4); // Check bluefruit.h for supported values

Bluefruit.Periph.setConnectCallback(connect_callback);

Bluefruit.Periph.setDisconnectCallback(disconnect_callback);

Bluefruit.setName(DEVICE_NAME);

bledis.setManufacturer("Adafruit Industries");

bledis.setModel("Bluefruit Feather52");

bledis.begin();

bleuart.begin();

blebas.begin();

blebas.write(100);

startAdv();

}

// Function to Start Advertisement

void startAdv(void) {

Bluefruit.Advertising.addFlags(BLE_GAP_ADV_FLAGS_LE_ONLY_GENERAL_DISC_MODE);

Bluefruit.Advertising.addTxPower();

Bluefruit.Advertising.addService(bleuart);

Bluefruit.ScanResponse.addName();

Bluefruit.Advertising.restartOnDisconnect(true);

Bluefruit.Advertising.setInterval(32, 244); // in unit of 0.625 ms

Bluefruit.Advertising.setFastTimeout(0); // number of seconds in fast mode

Bluefruit.Advertising.start(0); // 0 = Don't stop advertising after n seconds

}

// BLE Connected Callback Function

void connect_callback(uint16_t conn_handle) {

BLEConnection* connection = Bluefruit.Connection(conn_handle);

char central_name[32] = { 0 };

connection->getPeerName(central_name, sizeof(central_name));

Serial.print("Connected to ");

Serial.println(central_name);

dataflag = true;

}

/**

* Callback invoked when a connection is dropped

* @param conn_handle connection where this event happens

* @param reason is a BLE_HCI_STATUS_CODE which can be found in ble_hci.h

*/

// BLE Disconnected Callback Function

void disconnect_callback(uint16_t conn_handle, uint8_t reason) {

(void) conn_handle;

(void) reason;

Serial.println();

Serial.print("Disconnected, reason = 0x"); Serial.println(reason, HEX);

dataflag = false;

}

**// Sensor.cpp**

#include "main.h"

#include "sensor.h"

xQueueHandle queue;

float ax,ay,az,gx,gy,gz;

// Function to Initialize Sensor Task

void sensor_init(void){

imu.begin();

}

// Function to Run Sensor Task

void sensor_task(void *pvParam){

sensor_init();

TickType_t xLastWaketime;

const TickType_t xFrequency=100;

xLastWaketime = xTaskGetTickCount();

while(1){

// Read sensor data and store it in the buffer

ax = imu.readFloatAccelX();

ay = imu.readFloatAccelY();

az = imu.readFloatAccelZ();

gx = imu.readFloatGyroX();

gy = imu.readFloatGyroY();

gz = imu.readFloatGyroZ();

// Send current time to the queue

uint16_t ulvar = millis();

xQueueSend(queue, (void *)&ulvar, 0);

// Delay until the next execution

vTaskDelayUntil(&xLastWaketime, xFrequency);

}

}

**// Comm.cpp**

#include "main.h"

#include "comm.h"

#include "sensor.h"

#include <ArduinoJson.h>

char buf[96];

// Function to Initialize Communication Task

void comm_init(void){

printf("Comm Start");

}

// Function to Run Communication Task

void comm_task(void *pvParam){

comm_init();

while(1){

uint16_t ulvar;

xQueueReceive(queue, &(ulvar), portMAX_DELAY);

snprintf(buf, sizeof(buf),

"{\"ax\":%.2f,"

"\"ay\":%.2f,"

"\"az\":%.2f,"

"\"gx\":%.2f,"

"\"gy\":%.2f,"

"\"gz\":%.2f}\n",

ax, ay, az, gx, gy, gz

);

// sprintf(buf, "[%.2f,%.2f,%.2f,%.2f,%.2f,%.2f]",ax, ay, az, gx, gy, gz);

Serial.printf("%s\n",buf);

bleuart.write(buf);

}

}
