# Supplementary material for: The validity and reliability of motion analysis sensor system for wheelchair users (MASSWU)
Source: PLoS One. 2025 Oct 24;20(10):e0333391. doi: 10.1371/journal.pone.0333391 (PMC12551845; doi:10.1371/journal.pone.0333391)
Supplement: S2 File — (PDF) [file pone.0333391.s002.pdf]

ScF 03_01 (Eng)

The Human Research Ethics Committee of Thammasat University (Science), (HREC-TUSc) Room No 110, Piyachart Building, 1st Floor, Thammasat University Rangsit Campus, Prathumthani 12121 Thailand, Tel: 0-2564-4440 ext.7358 E-mail: ecsctu3@tu.ac.th

COA No. 080/2567

Certificate of Approval

Project No. : 67AH068
Title of Project : The Validity and Reliability of Motion Analysis Sensor System for Wheelchair Users
Principle Investigator : Sairag Saadprai
Place of Proposed Study/Institution: Faculty of Allied Health Sciences, Thammasat University

The Human Research Ethics Committee of Thammasat University (Science), Thailand, has approved the above study project in accordance with the compliance to the Declaration of Helsinki, the Belmont report, CIOMS guidelines and the International practice (ICH-GCP).

Signature: ....
(Assoc. Prof. Jinda Wangboonskul, Ph.D.)
Chairman of the Human Research Ethics Committee of Thammasat University (Science).

Signature: ....
(Assoc. Prof.Laksana Laokiat, Ph.D.)
Secretary of the Human Research Ethics Committee of Thammasat University (Science).

Date of Approval: July 23, 2024 **Approval Expire date:** July 22, 2025
Progressing Report Due: June 23, 2025

The approval documents

1) Research proposal Version 2/ 28-06-2024
2) Principal Investigator's Curriculum Vitae Version 2/ 28-06-2024
3) Patient/Participant Information Sheet Version 2/ 28-06-2024
4) Informed Consent Form Version 2/ 28-06-2024
5) Physical Fitness Tests for Wheelchair Users Version 2/ 28-06-2024
6) Physical Activity Readiness Questionnaire Plus Version 2/ 28-06-2024
7) Poster Version 2/ 28-06-2024
