# Supplementary material for: The validity and reliability of motion analysis sensor system for wheelchair users (MASSWU)
Source: PLoS One. 2025 Oct 24;20(10):e0333391. doi: 10.1371/journal.pone.0333391 (PMC12551845; doi:10.1371/journal.pone.0333391)
Supplement: Attachment — Submitted filename: Response to Reviewers.docx [file pone.0333391.s005.docx]

Dear Reviewers,

I hope this letter finds you well. I would like to express my sincere gratitude to you and the reviewers for your thoughtful comments and suggestions regarding my manuscript titled “The validity and reliability of motion analysis sensor system for wheelchair users (MASSWU)”. I have carefully considered each point raised and have made revisions accordingly. Below, I outline my responses to the reviewer’ comments point by point in the table below.

| **Reviewers’ comments** | **Edited sentences** |
| --- | --- |
| **Reviewer: 1**  Scientific Writing Quality: The manuscript suffers from poor English syntax, redundancy, and inconsistent terminology, which compromise clarity and scholarly tone. A thorough language revision by a native English speaker or a professional scientific editing service is strongly recommended. | The language throughout the document has been revised and enhanced. |
| Underperformance in the 360° Clockwise Rotation Test: The MASSWU sensor yielded only moderate reliability in the clockwise rotation task  (ICC = 0.712), in contrast to its performance in other tests. This may suggest a limitation in capturing rapid or complex rotational movements. Recommendation: The authors should provide a more in-depth technical analysis of this limitation. Could it stem from latency in gyroscope readings? Might the single-axis reference be compromised during unidirectional rotation? A potential solution could involve repositioning the sensor or employing a second sensor to enable cross-validation of rotational data. | Line 469  However, in terms of angular velocity measurement, in this study was found that for validity (360° CR Test) and for Intra-Class Correlation Coefficient (360° CR test and 360° AR test), MASSWU had only good validity (r=0.887) and good reliability (ICC=0.765, 0.865), possibly because the MASSWU sensor is mounted on the left wheel axle of the wheelchair to detect motion. MASSWU is a 6-axis IMU capable of measuring ax, ay, az, gx, gy, gz. The raw data from the sensor is processed to derive linear velocity and linear displacement. MASSWU, the gz axis is primarily used, as it lies in the same plane as the wheel’s rotation. When the wheelchair moves in a straight line, no centrifugal forces are involved, and tests under these conditions have shown no issues. However, in the 360° CR and 360° AR tests, the wheelchair is rotated in a circular path with the one of the wheels fixed as the pivot point and another wheel rotating clockwise and anticlockwise. This introduces centrifugal forces and motion components around the x and y axes, which contribute to measurement errors in the computed linear displacement. The computation principle is as follows: gz=angular velocity about the z-axis. Integrating gz over time yields the angular displacement about the z-axis. Multiplying this angular displacement by the wheel radius gives the linear displacement. If there is an error in orientation reconstruction from the IMU, the accuracy in assessing angular velocity will decrease, which in turn leads to the validity and reliability of the 360° CR test and 360° AR test not being at a very good level [44]. |
| Comparison With Similar Technologies: The discussion lacks a robust comparison with other validated IMU systems. A more critical contextualization of how MASSWU performs relative to existing inertial devices would strengthen the manuscript. | Line 437  Compared to other validated inertial measurement units (IMUs) commonly used in sports science and rehabilitation, such as the ActiGraph GT9X, Xsens MVN, or Noraxon MyoMotion systems, MASSWU’s performance is comparable in terms of reported correlation coefficients for movement parameters. For example, research using ActiGraph and Xsens devices has typically reported validity coefficients between 0.90-0.99 for distance, speed, and duration when benchmarked against reference standards during controlled wheelchair locomotion tasks [36-38]. While some advanced IMU systems may offer additional features, such as multi-segment kinematic analysis or integrated wireless synchronization for team environments, MASSWU’s validity aligns with or surpasses the core measurement accuracy reported in the literature for established IMU devices in wheelchair sport and mobility monitoring contexts. Nonetheless, it is important to note that, while different IMU platforms operate based on the same principles—utilizing accelerometers and gyroscopes—they may differ considerably in key aspects such as data acquisition and analysis techniques, calibration requirements, sensor placement flexibility, cost, battery life, and overall usability. These factors should be carefully considered alongside measurement validity when selecting an IMU system for specific research or practical applications. [39-40] |
| **Sample Characteristics and Generalizability:** The participant sample includes young adults (18–35 years old) with high functional capacity and no significant comorbidities, all of whom are habitual wheelchair users. While suitable for preliminary technical validation, this limits generalizability to broader clinical populations such as individuals with incomplete spinal cord injury, stroke, cerebral palsy, or other severe neurological impairments. **Recommendation:** The authors should explicitly acknowledge this limitation and expand the Discussion section to outline a concrete plan for future studies involving more clinically diverse and functionally representative cohorts. | Line 540  Finally, this study utilized a sample group aged 18-35 years, characterized by high physical capacity and no underlying medical conditions, making it suitable for assessing the validity and reliability of MASSWU. However, its applicability to broader populations remains limited. To address these limitations, the researchers plan to expand the sample size and diversity in future studies to include elderly individuals, as well as people with incomplete spinal cord injuries, stroke, cerebral palsy, and other severe neurological impairments, aiming to further develop and enhance the comprehensiveness of MASSWU. |
| Code and Algorithm Availability: The analytical algorithms and processing code used for the MASSWU data were not made available. This omission limits computational reproducibility. Recommendation: The authors are encouraged to share the code or provide supplementary material to ensure methodological transparency. | The authors have attached the document. |
| **Reviewer: 2**  Line 56-57, “Most sports scientists use the Video System for Motion Analysis, which is a standard method with both 2D and 3D analysis” – is it importante to cite a reference for this statement. | Line 56  Most sports scientists use the 2D and 3D Video System for Motion Analysis, which is a common tool used during sports training and competition to analyze movement [2]. |
| Line 99 - “This data is processed to calculate linear distance (based on wheelchair wheel size)  and speed. “–A more detailed description of how the data were processed would improve the understanding and potential reproducibility of the methodology used. | Line 94  The data acquisition and processing were performed on a XIAO nRF52840 Sense board equipped with an LSM6DS3TR-C inertial measurement unit (IMU) sampling at 10 Hz. The IMU provided 6-axis linear acceleration and angular velocity data (ax, ay, az, gx, gy, gz). In this study, the primary measurement for wheel rotation was the Z-axis angular velocity from the gyroscope (gz), which corresponds to rotation around the wheel’s central axis.  All signal processing and calculations were executed on the XIAO board using C code developed in the PlatformIO framework. The raw gz signal was first passed through an exponential moving average (EMA, α = 0.8) to attenuate high-frequency noise. The instantaneous linear velocity (v) was then computed directly from the filtered angular velocity according to equation (1), where r is the measured wheel radius:  $v\left( t \right)=gz,filtered\cdot r$ (1)  The total linear distance travelled s(t) was obtained by numerically integrating the velocity over time, as shown in equation (2), where Δt is the sampling interval:  $s\left( t \right)=s\left( t-1 \right)+v(t)\cdot\Delta t$ (2)  Each trial began with a 5-second static period to establish the baseline noise level. Movement onset was detected by the XIAO board when the absolute value of the filtered gz exceeded a threshold of three standard deviations above this baseline. Once movement was detected, the onboard software continuously calculated the average linear velocity, average angular velocity, duration, and total distance. Only these processed results were transmitted to the host computer via Bluetooth Low Energy (BLE), where they were displayed in real time and saved in Excel format. The user interface supported login, function selection, data collection, and real-time graph generation for intuitive interpretation. After data collection, the system automatically analysed the results and presented them in graphical form (Fig. 2) for easy visualization. |
| Line 109 – It seems that the entire paragraph is in the future, but the like would be in a project. The authors should describe how it hapends and the description of the individuals and if occurs dropouts. | Line 127  The participant selection for this research was based on specific qualifications: individuals had to be male or female, aged between 18 and 35 years, and daily wheelchair users. Participants were required to have the ability to propel their wheelchairs using their own arms and must not have had any serious underlying medical conditions such as heart disease, high blood pressure, or stroke. Additionally, participants could not suffer from severe muscle or nervous system pain that might interfere with their ability to participate in the study. The exclusion criteria included individuals scoring less than 9 on the Barthel Activities of Daily Living (ADL) scale[9], indicating limited capability for daily activities, and those who could not commit to the study period or had health concerns making physical activity unsafe, as assessed by the Physical Activity Readiness Questionnaire Plus (PAR-Q+)[10].  Participants who encounter difficulties during the research procedures—such as being unable to fully comply with the protocols or sustaining injuries during activities—are permitted to withdraw from the study at any time to safeguard their health and to maintain the integrity of the research data.  Throughout the study, the research team will closely monitor and document any potential physical or psychological risks to participants. If a participant is observed performing an incorrect posture or failing to follow the given instructions in a way that could cause harm—such as wheelchair overturning, falling from the wheelchair, or sustaining a muscle injury during testing—the researchers will immediately halt the activity to ensure maximum safety.  In the event of an injury, the researchers will assess the participant’s condition and provide initial first aid. If the condition does not improve, the participant will be referred for further medical treatment at Hospital, with emergency transportation arranged as necessary. The researchers will continue to monitor the participant’s condition and risk factors throughout the study to ensure both safety and peace of mind. Furthermore, participants have the right to withdraw from the research project at any time, without the need to provide a reason.  Each participant attended a single session lasting approximately three hours. Data collection took place at the indoor futsal field, Gymnasium 7, Thammasat University, Rangsit campus. |
| Line 129 – Since the final sample size differed from the initially estimated number of participants, a post hoc power analysis should be conducted to determine the actual statistical power achieved. Another point that requires clarification is why 23 individuals were assessed if the sample size calculation indicated that the test should be conducted with 15 participants. | Line 326  The initial sample size calculation using the G*Power program recommended 15 participants. However, since the target group was vulnerable and had a high chance of drop out, the researchers therefore kept in reserve for drop out of 100%, so the number of participants was increased to 30 participants.  Though, we screened 23 individuals to ensure we would achieve sufficient sample size. After applying the inclusion/exclusion criteria and considering consent withdrawals, 23 participants remained for analysis with no drop out. A post hoc power analysis was performed with the final sample of 23, using the same parameters (effect size=0.80, α=0.05), which demonstrated a statistical power of 0.956. This confirms that the study was adequately powered to detect large effects as originally intended.   |
| Line 183 – “The data from MASSWU and Kinovea were analysed to summarize the results thoroughly and accurately Details are needed regarding the analysis performed using Kinovea: which reference points were identified in the video for the calculation of the variables?.” | Line 223  Data Collection from MASSWU and Kinovea, for the first test, data from MASSWU and Kinovea were collected from the right wheelchair axle in the side view. Data recording began when the axle passed the floor markers placed by the researchers (serving as the starting reference point) and continued until the axle came to a complete stop (serving as the ending reference point). For the second test, data were also collected from the right wheelchair axle in the side view. Recording started when the axle passed the starting reference markers and ended when it passed another set of markers positioned 15 meters away (ending reference point). For the third and fourth tests, data were collected from the right wheelchair axle in the side view, starting as it passed the floor markers. Recording ended when the left wheelchair axle in the side view passed the same position used as the starting point. For the fifth and sixth tests, data were collected from both wheel axles in the front view. Recording began when both axles were positioned at the starting floor markers and ended when both returned to the same starting position twice.  Calibration for Kinovea Analysis, before analysing each test with Kinovea, the researchers placed two cones 20 meters apart on the floor. This distance served as a calibration reference to ensure measurement accuracy, and calibration was performed before each analysis session. |
| Line 212- Which variables presented a normal distribution and which did not? Following this analysis, which statistical tests were actually used for mean comparisons? | Line 350  Regarding the distribution of data in the validity test, the researchers found that the OSPT, 10X5MST, and ST tests exhibited a normal distribution; therefore, Pearson correlation and Paired T-Test analysis was employed. In contrast, the MPST, *360̊* CR, and *360̊* AR tests demonstrated a non-normal distribution; thus, the Spearman rank test and Wilcoxon Sign Rank Test was applied.  In the analysis of data distribution for the reliability tests, it was found that the data from the OSPT and ST tests were normally distributed; therefore, the Intra-Class Correlation Coefficient (ICC) using the Two-Way Mixed-Effects Model ICC (3,2) was chosen for analysis. In contrast, the data from the 10X5MST, MPST, 360̊ CR, and 360̊ AR tests were non-normally distributed; thus, Spearman rank correlation was used.  In the analysis of data distribution for the reliability test, it was determined that the OSPT and ST test data followed a normal distribution. Consequently, the Inter-Class Correlation Coefficient (ICC) with a Two-Way Mixed-Effects Model ICC (3,2), specifically ICC (3,2), was used. Conversely, the data from the 10X5MST, MPST, 360̊ CR, and 360̊ AR tests exhibited a non-normal distribution; therefore, the Spearman rank test was used. |
| Line 213 The description of the materials would be more coherently integrated at the end of the methodology section, followed by the description of the statistical analysis. | Line 192  The improvements have been made according to the recommendations. |
| Line 252 – “Research indicates this sensor has good validity (89.74%) in measuring fetal movement, as demonstrated in a 2023 study on wearable fetal movement detection (27)” – Explain how the results of Qin et al. (2023) could be applied or related to the context of your research.  In the tables, the data presented for the 360° CR and 360° AR tests show exactly the same means and standard deviations. This raises concern, and we would like to verify whether this is indeed correct or if there was a typographical error. | Line 303  Kinovea’s validity and reliability are comparable to 3D motion analysis programs [20-22] but Kinovea is better suited for field data collection [23-27]. It has served as a standard for validating devices [28-30]. Qin et al. (2023) evaluated a wearable fetal movement detection system using the NRF52840, which is the same core microcontroller as the XIAO nRF52840Sense sensor employed in our study. Their research demonstrated that, when configured for real-time movement detection and paired with appropriate accelerometers and algorithms, this hardware platform achieved an average recognition rate and correct rate of 89.74% for detecting fetal movements in a challenging real-world scenario—specifically, low-amplitude and subtle fetal motion in a clinical setting. This validation confirms the sensor's capability for accurate, reliable detection of small and complex human movements, even in environments that present high signal variability due to biological and environmental noise[31]. Although our research focuses on wheelchair movement, the technical demands are comparable-both require accurate real-time detection and differentiation of sensor signals corresponding to motion events of interest against potential background noise or artifacts. The high performance of the nRF52840-based system demonstrated by Qin et al. directly supports the suitability of using the XIAO nRF52840Sense sensor in our field setting, as it indicates the platform provides both the hardware reliability and signal fidelity necessary for precise human movement analysis.  The researchers examined the results of the 360̊ CR test and 360̊ AR test according to the reviewer’s suggestions. We found that the calculations were done incorrectly (originally calculated in units of s/degree), so we corrected the calculations to the proper units of degree/s. The researchers then recalculated, reanalyzed, and corrected the results, accordingly, as shown in Table 1-3; the results of the 360̊ CR test and 360̊ AR test. |
| The Discussion section could better relate the findings to the existing literature by citing studies with similar results that reported high correlation coefficients, and further explore the reasons behind these findings. | Line 418  The Discussion section has been revised to better relate the findings to the existing literature by incorporating citations of studies reporting similarly high correlation coefficients. |
| 452 - In the conclusion, the results may not sufficiently support such extensive extrapolation, given that conditions in wheelchair sports competitions and training are highly variable and involve greater distances and different coverage areas, which could lead to different sensor readings. Since the evaluation was conducted under specific tests, the conclusion should be limited to what was actually assessed, and any extrapolations should be made with appropriate caution." | Line 550  This study found that the MASSWU demonstrated good to very good validity compared with Kinovea, along with good to very good intra- and inter-rater reliability. These results indicate that MASSWU can accurately detect, record, and analyze wheelchair motion parameters—such as distance, duration, speed, and angular velocity—across the six-wheelchair skill-related fitness tests (OSPT, MPST, 10×5MST, ST, 360° CR, and 360° AR) employed in this study. While these findings are promising, it should be noted that conditions in wheelchair sports competitions and training can vary considerably in terms of distance, movement patterns, coverage area, and the diversity of impairments among athletes, all of which may influence sensor performance. Therefore, any application of these findings to real-world settings should be made with caution, and further validation under competitive and training conditions, as well as in groups with different types of impairments, is recommended. |

We believe these revisions have strengthened the manuscript and addressed all concerns raised by the reviewers. Thank you once again for your valuable feedback. We look forward to your response and hope for a favorable consideration of our revised manuscript.

Sincerely,
